# Assessment of *Chlorella sorokiniana* Growth in Anaerobic Digester Effluent

**DOI:** 10.3390/plants10030478

**Published:** 2021-03-03

**Authors:** Elvira E. Ziganshina, Svetlana S. Bulynina, Ayrat M. Ziganshin

**Affiliations:** Department of Microbiology, Institute of Fundamental Medicine and Biology, Kazan (Volga Region) Federal University, 420008 Kazan, Russia; elvira.ziganshina@kpfu.ru (E.E.Z.); SvSBulynina@stud.kpfu.ru (S.S.B.)

**Keywords:** photobioreactor, anaerobic digester effluent, microalgal–bacterial polyculture, *Chlorella sorokiniana*, bacterial community structure, nutrient removal

## Abstract

Microalgae are considered a potential source of valuable compounds for multiple purposes and are potential agents for bioremediation of aquatic environments contaminated with different pollutants. This work evaluates the use of agricultural waste, unsterilized and anaerobically digested, to produce biomass from a strain of *Chlorella sorokiniana*. Furthermore, the presence of bacteria in these wastes was investigated based on the bacterial 16S rRNA gene sequencing. The results showed a specific growth rate ranging between 0.82 and 1.45 day^−1^, while the final biomass yield in different digestate-containing treatments (bacterial-contaminated cultures) ranged between 0.33 and 0.50 g L^−1^ day^−1^. Besides, substantial amounts of ammonium, phosphate, and sulfate were consumed by *C. sorokiniana* during the experimental period. The predominant bacteria that grew in the presence of *C. sorokiniana* in the effluent-containing treatments belonged to the genera *Chryseobacterium*, *Flavobacterium*, *Sphingomonas*, *Brevundimonas*, *Hydrogenophaga*, *Sphingobacterium*, and *Pseudomonas*. Therefore, this microalga can tolerate and grow in the presence of other microorganisms. Finally, these results show that anaerobically digested agricultural waste materials are a good substitute for growth media for green microalgae; however, phosphate and sulfate levels must also be controlled in the media to maintain adequate growth of microalgae.

## 1. Introduction

Microalgae are deservedly considered promising renewable sources of various valuable compounds—proteins, lipids, pigments, antioxidants, as well as valuable food and feed additives. Microalgae are not only a potential source of nutrients and biologically active compounds for use in the food and pharmaceutical industries but also attract the attention of researchers because of their ability to reduce greenhouse gas emissions and remove inorganic nitrogen, phosphorus, heavy metals, and some toxic organic compounds [1,2,3,4]. However, despite the excellent prospects for microalgal biotechnologies, the cost of production and processing of microalgal bioproducts is often a limiting factor for their large-scale implementation. Thus, microalgal biotechnology requires inexpensive water and nutrients for high and stable algal growth rates and increased target products’ productivity.

Environmental pollution by various compounds is a big problem everywhere [5,6]. Considering the need to address environmental pollution issues and maintain water resource availability and quality, biological wastewater treatment using microalgae is rapidly developing as an economically and ecologically attractive biotechnology. Many studies to optimize the growth and productivity of microalgae under various regimens have focused on growing microalgae in different nutrient-rich wastewater streams, including municipal [7,8], industrial [9,10,11], and agricultural wastes [12,13]. It seems possible to use microalgae for the simultaneous achievement of several goals: wastewater treatment, synthesis of valuable metabolic products, and accumulation of algal biomass as feed supplements. Various waste streams that are used as a nutrient medium for growing promising microalgae, in addition to valuable nutrients, may contain heavy metals, metalloids, pathogens, and various organic pollutants. The researchers concluded that a few resistant strains of algae could grow and efficiently produce valuable bioproducts in such harsh conditions, given the fact that these substrates can contain large amounts of chemical contaminants and other microorganisms [12,13,14,15].

Agricultural wastewater contains different microorganisms that are usually excreted in the feces and urine of farm animals. The abundance and diversity of these microorganisms are the subjects of distinct studies, since their presence in wastewater directly affects the growth rate and productivity of algal cultures, as well as the quality of products obtained from algal biomass. Bohutskyi with colleagues [12,13] proved that only a few algae of the genera *Chlorella* and *Scenedesmus* could grow efficiently in the bacterial-contaminated wastewater media. *Chlorella* species, such as *Chlorella vulgaris* and *Chlorella sorokiniana*, are admirable for autotrophic, heterotrophic, and mixotrophic growth. They are actively used for wastewater treatment alone [15,16,17] or in combination with activated sludge [14,18,19]. Moreover, they belong to the producers of valuable biologically essential compounds such as pigments, lipids, proteins, and carbohydrates [20,21]. Furthermore, extracts of microalgae of the genera *Chlorella* and *Scenedesmus* positively affect the germination of root crops [22].

Researchers in this area pay great attention to optimizing the nutritional conditions in order to increase the biomass yield of these photosynthetic microorganisms and the productivity of individual products of their metabolism. A distinctive feature in this area is the search and use of cheap media to cultivate algae. These include wastewater and some products of processing various organic waste materials, particularly wastes generated during the anaerobic digestion process [23,24]. Anaerobic digestion of biomass is a standard process applied to treat a wide range of organic waste materials. Complex microbial communities carry out this process with simultaneous biogas production [25,26]. In addition to biogas, the gaseous product of the anaerobic process, effluents (digestates) are generated, which are rich in nitrogen and phosphorus compounds. They can be considered as inexpensive and suitable media for growing microalgae [16,23]. Thus, vast new data on the screening, characterization, and efficient cultivation of green microalgae using affordable and nutrient-rich substrates could significantly increase microalgae productivity for the food, pharmaceutical, and biofuel industries. In our recent work [27], we optimized the nutrients levels and light intensity for the high growth rate of alga *C. sorokiniana* AM-02 in photoautotrophic growth regimens. Thus, a wide range of nitrate levels (180–1440 mg·L^−1^) and different photosynthetic photon flux density conditions (1000–1400 μmol·m^−2^·s^−1^) were tested on the growth efficiency of *C. sorokiniana* AM-02. We further suggested that this local strain is suitable for enhanced biomass productivity and purification of different wastewater systems.

In this work, we evaluated the growth parameters of *C. sorokiniana* strain AM-02, as a strain resistant to high concentrations of nutrients and high light intensity, during its cultivation in an unsterilized anaerobic digestion effluent. Growth, the concentration of pigments, pH of the medium, utilization of nutrients by microalgal culture were investigated throughout the entire experimental period. The optimal cultivation conditions for the effective removal of nutrients were identified. Besides, bacterial 16S rRNA gene fragments were examined to analyze the level of bacterial contamination in the media.

## 2. Results and Discussion

### 2.1. Growth of C. sorokiniana AM-02 under Different Conditions

The growth of microalgae and biomass productivity when grown in wastewater or anaerobic digester effluent depend on different factors (such as the features of the culture, physicochemical properties of the wastes, type of a photobioreactor, and technological parameters of the process) [28]. The microalgal strain *Chlorella sorokiniana* AM-02 was tested in our previous study [27], in which we identified the optimal growth conditions in a standard Bold’s Basal Medium (BBM). In this research, a synthetic medium for supplying algae with all the necessary growth compounds was replaced with a diluted anaerobic digester effluent (ADE). Unsterilized effluent after mesophilic anaerobic digestion of cattle manure, distiller grains with solubles, and sugar beet pulp was used for the experiments. ADE was initially centrifuged at 10,000× *g* for 10 min to remove sediment and then was used to culture *Chlorella sorokiniana* strain AM-02.

**Figure 1:**
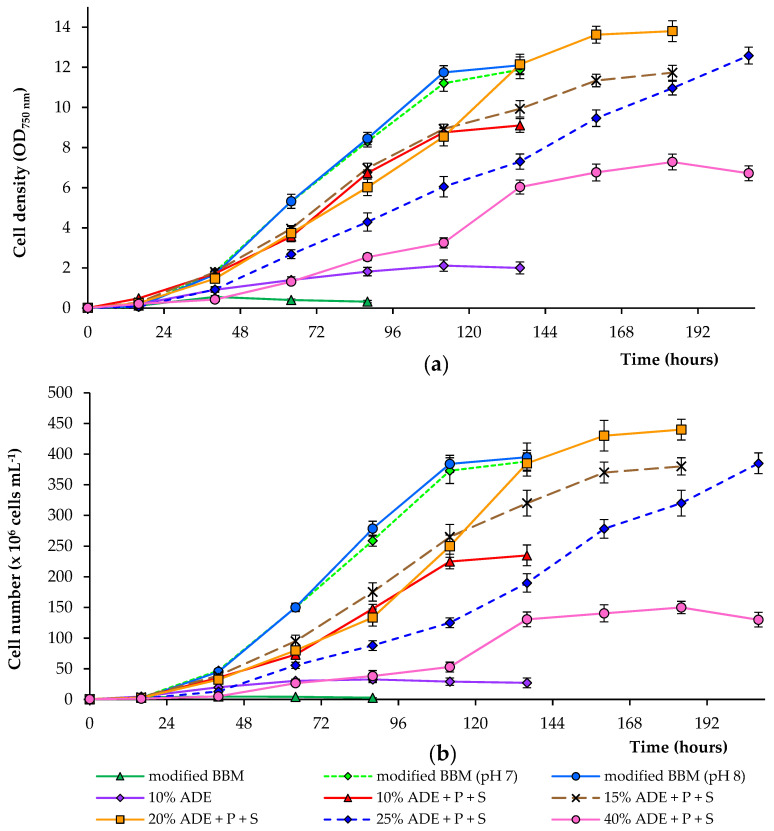
Growth of *C. sorokiniana* AM-02 (OD_750_ (**a**) and cells·mL^−1^ (**b**)) cultured under different conditions.

We studied the effect of different concentrations of digestate (10, 15, 20, 25, and 40%) on the growth of the alga (ADE was diluted in deionized water). These wastes are rich in nitrogen and contain residual organic carbon, which can be used by microalgae. Most of the nitrogen in the digestate was in the form of ammonium and was readily available for algae. It is important to note that high ammonium concentrations are toxic for various microalgae, and ammonium toxicity in water can be due to non-ionized ammonia (NH_3_) and ionized ammonium (NH_4_^+^). NH_3_ is considered the most toxic form for different microalgae, because it is lipid-soluble and diffuses readily through membranes [29]. Besides, a modified BBM with an ammonium nitrogen source was also used to compare growth efficiency. To control the algal growth, the OD_750_ was measured. Since OD_750_ also measures bacterial growth, the number of algal cells was further counted (results are shown in Figure 1). The algae were cultured until the stationary phase was reached.

During the growth of alga *C. sorokiniana* in 10% ADE, we noted that, among various other factors, a significant factor is the presence of available phosphate and sulfate ions in diluted ADE. An initial experiment showed that due to a lack of these compounds in diluted effluent, the growth of *C. sorokiniana* strain AM-02 was limited. After adding phosphate (up to 160 mg·L^−1^) and sulfate (up to 40 mg·L^−1^) to the diluted effluent, all critical parameters were significantly improved (Figure 1; Table 1 and Table 2). Higher growth and biomass production were observed when the medium contained 10–40% digestate with additional phosphate and sulfate ions.

Adding phosphate and sulfate and increasing the concentration of ADE in the culture medium from 10 to 20% led to an increase in OD_750_ and cell numbers. Overall, OD_750_ correlated with the calculated cell count. The optical density changes were also due to bacterial cells’ growth on effluent components. Still, the main reason for OD_750_ change was the growth of microalgae, since the abundance of bacteria was low compared with the abundance of algal cells (data not shown). The cultures in experiments supplied with 10% ADE increased in mean OD_750_ values and cell number until stationary growth was achieved at 8.9 and 2.3 × 10^8^ cells·mL^−1^ (after 112 h), respectively. In comparison, in treatments supplied with 20% ADE, the cultures increased in mean OD_750_ values and cell number to 13.6 and 4.3 × 10^8^ cells·mL^−1^ (after 160 h), respectively.

Further expanding the ADE concentration in the culture medium from 20 to 40% led to a decrease in mean OD_750_ values and cell numbers compared to the previous experiments. The cultures in treatments supplied with 25% ADE reached mean OD_750_ values and cell number at 12.6 and 3.9 × 10^8^ cells·mL^−1^ (after 208 h), respectively. In contrast, in treatments supplied with 40% ADE, the cultures achieved mean OD_750_ values and cell number until stationary growth was achieved at 7.3 and 1.5 × 10^8^ cells·mL^−1^ (after 184 h), respectively. Cultivation in modified BBM (with controlled pH levels of 7.0 and 8.0) was similar but showed better growth characteristics compared to those observed in diluted effluents. Cultivation in modified BBM without pH control resulted in a rapid drop in the medium’s pH and complete growth inhibition (Figure 1).

Key parameters of the different growth conditions, including dry weight, volatile solids, specific growth rate, biomass productivity, and maximum pigments, were determined, and these data are displayed in Table 1 and Table 2. *C. sorokiniana* strain AM-02 grown in 10% ADE without and with the addition of phosphate and sulfate reached maximal mean biomass concentrations of 0.44 and 1.88 g·L^−1^, respectively. These data were significantly lower than the values obtained in modified BBM (2.8 g·L^−1^). *C. sorokiniana* AM-02 maintained in 20 and 25% ADE with the addition of phosphate and sulfate reached a maximal mean biomass concentration of 3.35–3.39 g·L^−1^. These values were higher than the values observed in modified BBM, but statistically insignificant. During the first days, a high foam generation started in the photobioreactor with 40% effluent loading (despite the addition of antifoam). Higher levels of dry matter in these treatments can be explained by the immobilization of algal cells and the formation of biofilms on the reactor’s inner surfaces in the air phase (after the foam level decreased), which were also collected after the experiment.

The mean specific growth rates of *C. sorokiniana* strain AM-02 were 1.45, 1.06, 1.08, 0.82, and 0.86 day^−1^ in 10% ADE, 15% ADE, 20% ADE, 25% ADE, and 40% ADE with the addition of phosphate and sulfate, respectively. However, these values were significantly lower than those values observed during the growth of *C. sorokiniana* in modified BBM (1.50–1.51 day^−1^). The highest growth rate of 1.45 day^−1^ was obtained with 10% effluent loading compared to other ADE-containing treatments. The mean biomass productivities were 0.40, 0.41, 0.50, 0.39, and 0.33 g·L^−1^·day^−1^ in 10% ADE, 15% ADE, 20% ADE, 25% ADE, and 40% ADE with the addition of phosphate and sulfate ions, accordingly. Values observed in ADE-containing treatments were significantly lower than those observed during *C. sorokiniana* cells’ growth in modified BBM (0.60 g·L^−1^·day^−1^). The highest biomass productivity of 0.50 g·L^−1^·day^−1^ was obtained with 20% effluent loading compared to other ADE-containing experiments.

**Figure 2:**
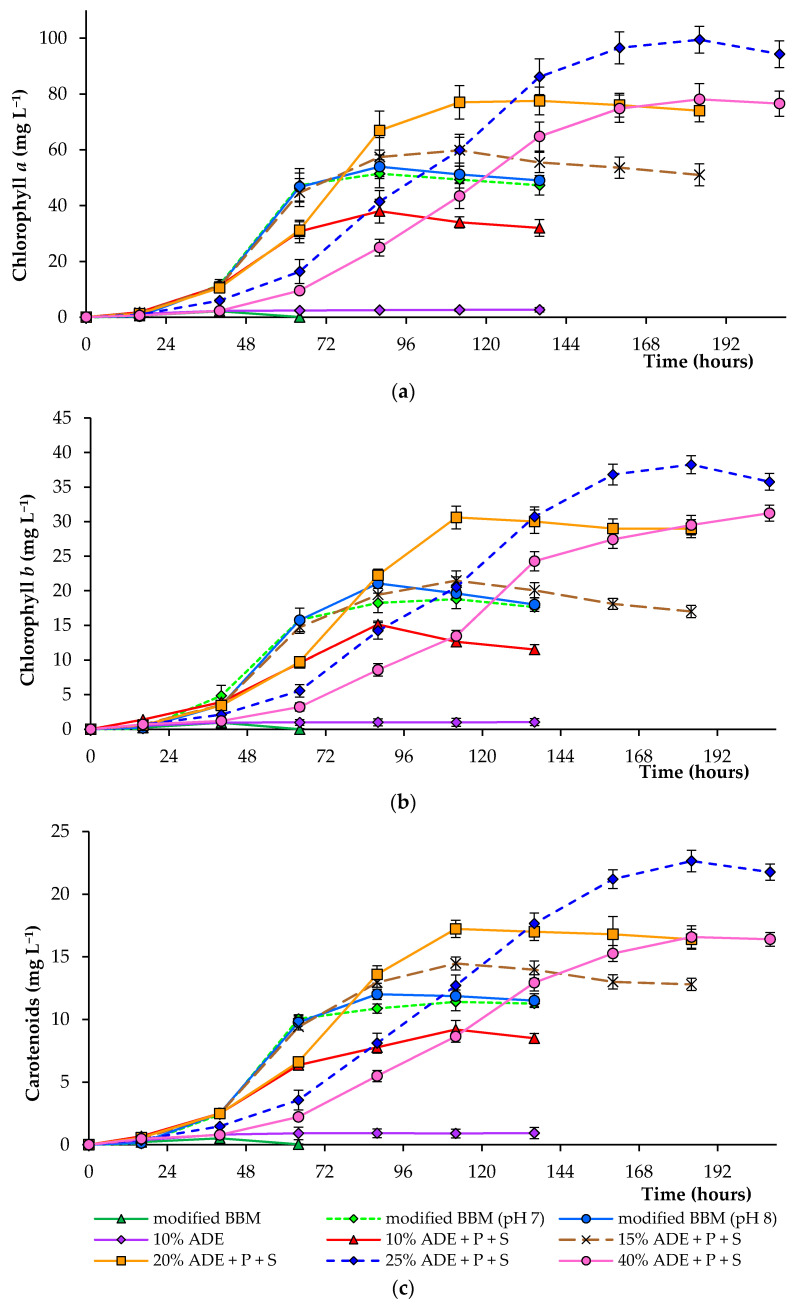
Pigment concentration (chlorophyll *a* (**a**), chlorophyll *b* (**b**), and carotenoids (**c**)) in cells of *C. sorokiniana* AM-02 cultured under different conditions.

Figure 2 demonstrates the concentrations of chlorophyll *a*, chlorophyll *b*, and total carotenoids in algal cells cultured under various experimental conditions. Thus, the final pigment concentration in algal cells cultured in 20–40% ADE was significantly higher than that observed in cells cultured in modified BBM (Table 2). Culturing of alga in 25% ADE resulted in the highest pigment concentration, and the mean chlorophyll *a*, chlorophyll *b*, and total carotenoids reached 99.5, 38.3, and 22.7 mg·L^−1^ under these conditions, respectively. The mean chlorophyll *a*, chlorophyll *b*, and total carotenoids reached 51.4, 18.2, and 10.9 mg·L^−1^ during culturing in modified BBM, accordingly (at pH 7.0) (Figure 2).

The slower growth of different green microalgae in various agricultural wastewaters is also due to their dark color. The high level of total solids in different digestates and the intense black color of non-diluted effluents reduce light penetration into the culture medium and, thereby, hinder microalgal growth and reduce the rate of nutrient recovery from wastewaters as was shown previously [30,31]. According to previous studies, the medium’s dark color lowers the algal cellular productivity compared to a non-colored culture medium [17,31,32]. 

This research showed that microalgae’s successful growth in diluted anaerobic digester effluent was believed to be due to the improved light transmission and reduced ammonia (and possibly other components) toxicity. In several research works, light limitation and ammonia toxicity have been avoided by diluting the wastewater with clean water [17,32,33]. Centrifugation of the digestate contributed to removing particles as well, which gave it a dark color.

Previous work results showed that the preferred high levels of photosynthetic photon flux density for *C. sorokiniana* strain AM-02 in standard BBM are 1000–1400 μmol of photons m^−2^·s^−1^ [27]. Therefore, we chose 1200 μmol of photons m^−2^·s^−1^ in the present study. Under such conditions, microalgae should receive enough light, since the addition of digestate increases the medium’s turbidity. Moreover, we continuously sparged cultures with air supplemented with 2% CO_2_, since it was found that nutrients are consumed faster under these conditions [27].

**Figure 3:**
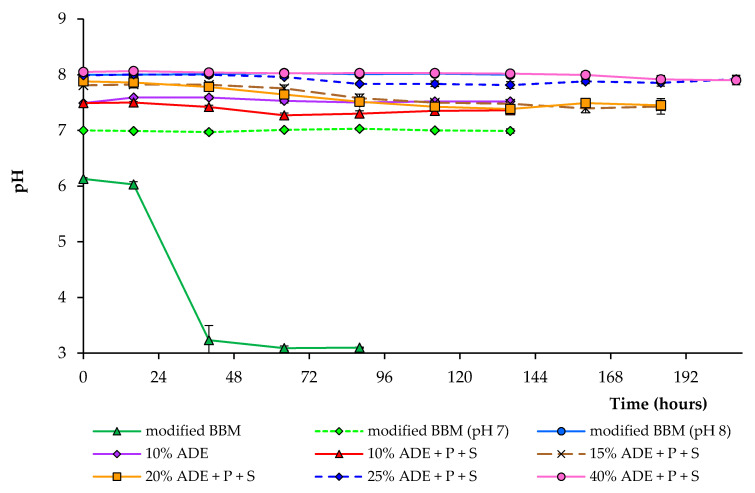
pH changes during the growth of *C. sorokiniana* AM-02 under different conditions.

Figure 3 shows the changes in pH under different cultivation conditions. The higher ADE content resulted in a higher initial pH of the medium. During cultivation in various diluted anaerobic digester effluents, the pH decreased from initial values of 7.5–8.0 to about 7.3–7.9, depending on the experimental condition. In the experiments with modified BBM, pH dropped from an initial 6.1 to 3.2 within 40 h during the cultivation of *C. sorokiniana* strain AM-02, which completely inhibited the alga growth. Ammonium nutrition leads to a liberation of H^+^ and a decrease in the medium’s pH [29]. Under pH-controlled conditions, growth characteristics in modified BBM were utterly identical, regardless of pH 7.0 or 8.0 (Figure 3).

**Figure 4:**
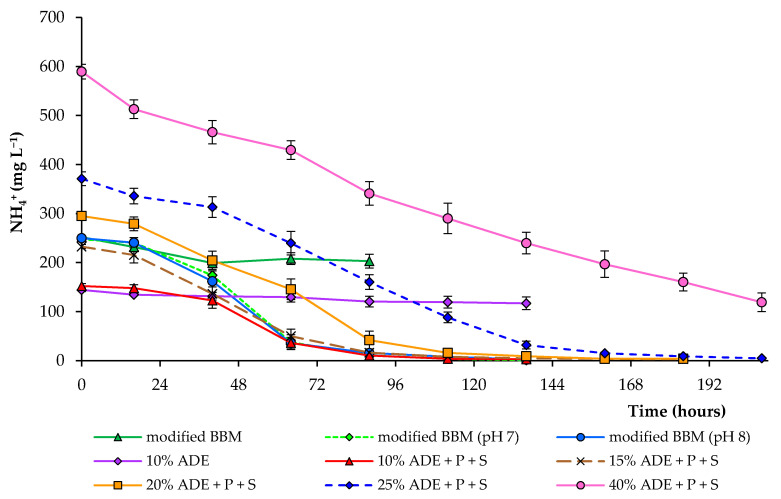
Ammonium concentrations in modified BBM and diluted ADE during the growth of *C. sorokiniana* AM-02.

Various nitrogen sources in nutrient media promote the growth of microalgae [34,35,36,37]. At non-toxic concentrations, ammonium has been reported to induce higher growth rates than nitrate and urea for many different microalgae species [29]. As previously indicated, the ammonium removal efficiency varies depending on the media composition and environmental conditions, such as the initial concentration of nutrients, light intensity, light/dark cycle, as well as algae species [38]. Most of the nitrogen in the digestate in this study was in the form of an ammonium. Comparison of ammonium removal during the entire experimental period at different digestate concentrations is shown in Figure 4.

In this study, the initial values of the ammonium content in the medium varied depending on the treatment. When cultivating algal cells in a medium loading with 10% digestate, only 17% of ammonium was removed after 112 h. Still, when additional nutrients were added to the growth medium, 99% of ammonium was removed after 112 h. All subsequent experiments were carried out with the addition of phosphate and sulfate at the same concentration. A similar trend was observed using 15, 20, and 25% diluted ADE (99% removal after 136–184 h). With an increase in ADE loading to 40%, ammonium removal reached 80% after 208 h. The rapid NH_4_^+^ removal efficiency was achieved after 88–112 h in modified BBM (pH-controlled conditions) and 10–20% ADE (with added phosphate and sulfate ions). Our results also show that a high phosphate and sulfate removal level is possible (Table 1). However, the partial removal of phosphate in ADE-containing treatments could also be due to some phosphate minerals’ precipitation. These findings indicate that *C. sorokiniana* AM-02 is incredibly tolerant to high ammonium levels. Hence, it has a potential role in removing high amounts of ammonium, phosphate, and sulfate from wastewater. However, an additional contribution to the removal of nutrients in ADE-containing treatments was made by bacteria that grew in the presence of *C. sorokiniana*.

Even though the abundance of various essential compounds in different wastewaters and anaerobic digesters effluent makes it suitable for microalgae cultivation, it contains other microorganisms, which can compete with microalgae cells. Therefore, most studies cultivated microalgae in sterilized wastewater [11,36,38]. However, various sterilization methods can increase the cost of culturing of microalgae and are economically impractical for large-scale cultivation. In this research, ADE was pretreated by centrifugation to remove sediment and improve light transmission (however, for large-scale cultivation, other methods should also be considered). This method also removed most of the microorganisms; however, a minor part was still present in pretreated ADE. The influence of various microorganisms on the growth of *C. sorokiniana* AM-02 was studied in such an environment to develop effective methods for large-scale cultivation of microalgae in multiple wastewaters. It is imperative to screen for resistant microalgae species that can tolerate and grow effectively in such environments, removing nutrients and producing biomass, and in the presence of other competing microorganisms.

Among the studies describing microalgae cultivation in such wastewater systems, the following examples should be mentioned. Bohutskyi et al. [13] found that *C. sorokiniana* and *Scenedesmus acutus* are characterized by higher growth rates, productivity, and resistance than other microalgal species when grown in diluted wastewaters samples. Chen et al. [15] demonstrated that the highest biomass concentration (5.45 g·L^−1^) by *C. sorokiniana* AK-1 could be obtained after 15 days when maintained in 50% strength swine wastewater, preliminary pretreated by filtration to remove sediments. Kobayashi et al. [16] investigated the growth of three *Chlorella sorokiniana* strains in 10% anaerobic digester effluent obtained from cattle manure digestion. ADE was pretreated by centrifugation in their research. Biomass was produced at a concentration of about 270 mg·L^−1^ by the strains UTEX 1230 and CS-01 (after 21 days) but inhibited the growth of the strain UTEX 2714 by more than 50% in the ADE. Lizzul et al. [36] found that the final biomass yield of *C. sorokiniana* UTEX1230 cultured in different wastewater samples that were autoclaved and diluted to 10% ranged between 220 and 320 mg·L^−1^. However, the bacterial community structure in these experiments was not investigated. In our work, we show higher biomass productivity compared to the results mentioned above. Our results indicate that *C. sorokiniana* AM-02 is a good candidate for simultaneous wastewater treatment and biomass production.

### 2.2. Bacterial Community Structure

Algal systems consume nutrients more efficiently and provide oxygen to the aerobic bacteria [39]. Some studies demonstrate the possibility of different symbiotic relationships between microalgae and bacteria. These relationships’ nature is still mostly unknown, but there is evidence that most of such relationships allow algae and bacteria to exchange essential metabolites. Although the possibility of developing close relationships in different growth systems has been previously shown, there is little research regarding the impact of these relationships on biomass productivity or the ability to occupy a specific ecological niche [40].

Five samples were taken from several of our ADE-containing systems to analyze bacterial communities’ structure developed during the experimental period (at last days). More than 400,000 high-quality bacterial sequences were obtained, and the average number of reads per sample was 80,368 (from 65,191 to 94,835). In general, sequencing of amplicons covered most bacterial phylotypes, which were observed in five samples. The alpha diversity indices (operational taxonomic units (OTUs), Chao1 index, Shannon index, Simpson index) calculated on the OTU level for each sample are demonstrated in Table 3. The number of bacterial OTUs in five samples ranged from 55 to 61 (abundance > 0.1%), and their number was comparable in all ADE-containing treatments. However, their abundance was low in comparison with algal cells (data not shown).

**Figure 5:**
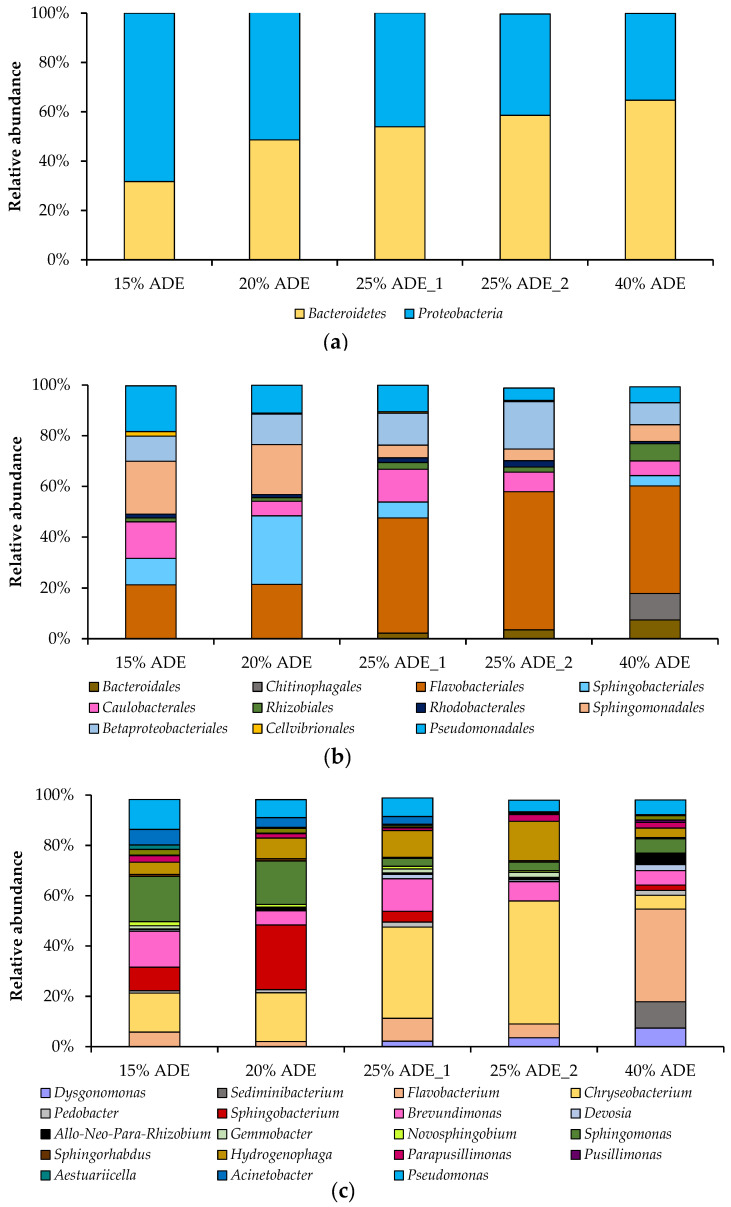
Taxonomic composition of bacterial communities in the bioreactor during *C. sorokiniana* AM-02 cultivation in ADE-containing media (15% ADE (184 h), 20% ADE (184 h), 25% ADE_1 (160 h), 25% ADE_2 (208 h), and 40% ADE (208 h)). Bacterial community composition according to amplicon sequencing of the bacterial 16S rRNA gene is shown on the phylum (**a**), order (**b**), and genus (**c**) levels. Only genera with a relative abundance of at least 1% in at least one sample are shown.

The relative abundance of different bacteria that grew in the presence of *C. sorokiniana* AM-02 has been investigated on different taxonomic levels, such as phylum, class, order, family, and genus. Thus, two phyla, three classes, eleven orders, sixteen families, and twenty-six genera were detected in five samples. The structure of bacterial communities (on the phylum, order, and genus levels) in different treatments is presented in Figure 5. The predominant bacterial phyla in the treatments containing ADE were *Bacteroidetes* and *Proteobacteria*, which accounted for 51 and 49% of the total bacterial 16S rRNA gene sequences, respectively (Figure 5a). The predominant bacterial orders in the ADE-containing treatments were *Flavobacteriales*, *Betaproteobacteriales*, *Sphingomonadales*, *Sphingobacteriales*, *Pseudomonadales*, and *Cauobacterales*, which accounted for 37, 12, 11, 10, 10, and 9% of the total bacterial 16S rRNA gene sequences, accordingly (Figure 5b). The relative abundance of members belonging to the phylum *Proteobacteria* decreased, while the relative abundance of representatives affiliated with the phylum *Bacteroidetes* increased with ADE concentration (Figure 5a).

The predominant bacterial genera in the treatments loaded with 15% ADE were *Sphingomonas*, *Chryseobacterium*, *Brevundimonas*, *Pseudomonas*, and *Sphingobacterium* (sampled on 184 h). The prevailed bacterial genera in 20% ADE-containing treatments were *Sphingobacterium*, *Chryseobacterium*, *Sphingomonas*, *Hydrogenophaga*, and *Pseudomonas* (sampled on 184 h). The predominant genera in 25% ADE treatments were *Chryseobacterium*, *Brevundimonas*, *Hydrogenophaga*, *Flavobacterium*, and *Pseudomonas* (sampled on 160 h (ADE_1) and 208 h (ADE_2)). The predominant genera in 40% ADE-containing experiments were *Flavobacterium*, *Sediminibacterium*, *Dysgonomonas*, *Brevundimonas*, *Sphingomonas*, and *Pseudomonas* (sampled on 208 h) (Figure 5c).

Members of the genus *Sphingomonas* are aerobic bacteria that are ubiquitous in the environment, including water, soil, and activated sludge. Sphingomonads are used for a wide range of biotechnological applications, from the bioremediation of environmental pollutants to the production of extracellular polymers for food and other industries [41]. Species of the aerobic genus *Sphingobacterium* have mainly been isolated from soil, compost, sludge, plants, raw milk, and water [42]. They are also involved in biodegradation processes [43]. *Brevundimonas* species are aerobic and are widespread in the environment, including soils, activated sludge, aquatic habitats, and clinical specimens [44]. Some of them are considered potential candidates for remediation of sites contaminated with diesel, n-alkanes, and polycyclic aromatic hydrocarbons [45,46]. Species of the *Chryseobacterium* are aerobic and producing flexirubin pigments, which give the colonies a light yellow or yellowish-orange color. They can be isolated from various habitats and involved in remediation processes [47]. *Pseudomonas* spp. are aerobic and excellent bacteria for use in bioremediation processes due to the flexibility and plasticity of their metabolic pathways [48]. *Hydrogenophaga* species possess oxidative metabolism and can be isolated from activated sludge and wastewater systems [49]. Members of the genus *Flavobacterium* are aerobic and distributed widely in nature [50]. Besides, bacteria that are used for bioremediation processes include members of the genera *Pseudomonas*, *Sphingomonas*, *Chryseobacterium*, as well as *Flavobacterium* [51]. Most of these bacteria were also detected as main representatives of microbial communities in other algae–bacteria systems that grew in other wastewater systems [52] and freshwater reactors [53]. Interestingly, representatives of the orders *Rhizobiales*, *Betaproteobacteriales*, and *Chitinophagales* correlated with biomass productivity of *C. sorokiniana* strain DOE1412 during outdoor cultivation [53].

Bacteria from biogas reactors are mostly strictly anaerobes and facultative anaerobes, and the small part of these anaerobic bacteria that remained after the pretreatment of ADE was quickly replaced by aerobic microflora (still present in effluent and from different sources during effluent preparation) during algae growth. Some bacteria that grew in the presence of *C. sorokiniana* AM-02 were also found in the initial effluent [54,55]. It can be assumed that these bacteria, better than other microbes, adapted to the constructed environment, and some of them favorably coexisted with microalgae cells, for example, during the acquisition and exchange of essential metabolites. The investigation of the microbial community structure in algal/bacterial systems provides a necessary insight into various water body systems that can be used to control algal biomass productivity and algal health in non-sterile environments.

## 3. Materials and Methods

### 3.1. Digestate-Based Media Preparation

Effluent from mesophilic batch digesters after anaerobic digestion of cattle manure, rye distiller’s grains with solubles, and sugar beet pulp was selected to test the growth of the microalga. Initially, the anaerobic digester effluent (ADE) was centrifuged at 10,000× *g* for 10 min to remove sediment, diluted with deionized water to different concentrations (10–40% *v/v*), and then used to cultivate *Chlorella sorokiniana* strain AM-02. In experiments with the addition of phosphate ion and sulfate ion, K_2_HPO_4_ and H_2_SO_4_ were added to the diluted effluent to reach standard concentrations as in Bold’s Basal Medium (BBM) (~160 mg·L^−1^ and ~40 mg·L^−1^ for phosphate and sulfate ions, respectively). Before the start of the experiments, ADE was stored at +4 °C.

Digestate for all treatments was initially analyzed to determine pH, total solids (TS), volatile solids (VS), and concentrations of total volatile fatty acids (VFA), as described in detail previously [54,55]. Phosphate and sulfate concentrations in the digestate were analyzed as described before [27]. Thus, initial ADE had the TS content of 4.9 ± 0.14%, VS content of 3.6 ± 0.11%, pH of 8.0 ± 0.05, and VFA concentration of 0.54 ± 0.05 g·L^−1^. Pretreated 100% ADE had initial phosphate and sulfate levels of 52.3 ± 1.6 mg·L^−1^ and 5.7 ± 0.7 mg·L^−1^, respectively. All these parameters were measured in triplicate, and the mean values are presented together with the standard deviations.

### 3.2. Cultivation Conditions in a Photobioreactor

*C. sorokiniana* AM-02 was isolated from a local freshwater lake, and the characteristics of its photoautotrophic growth and biomass productivity in BBM were described previously [27]. Microalga was maintained on the plates with standard BBM [56], supplemented with kanamycin (50 μg·mL^−1^) and ampicillin (10 μg·mL^−1^). All manipulations were performed under sterile conditions to avoid contamination. Soil extract and vitamin mix were not added to the original BBM.

**Figure 6:**
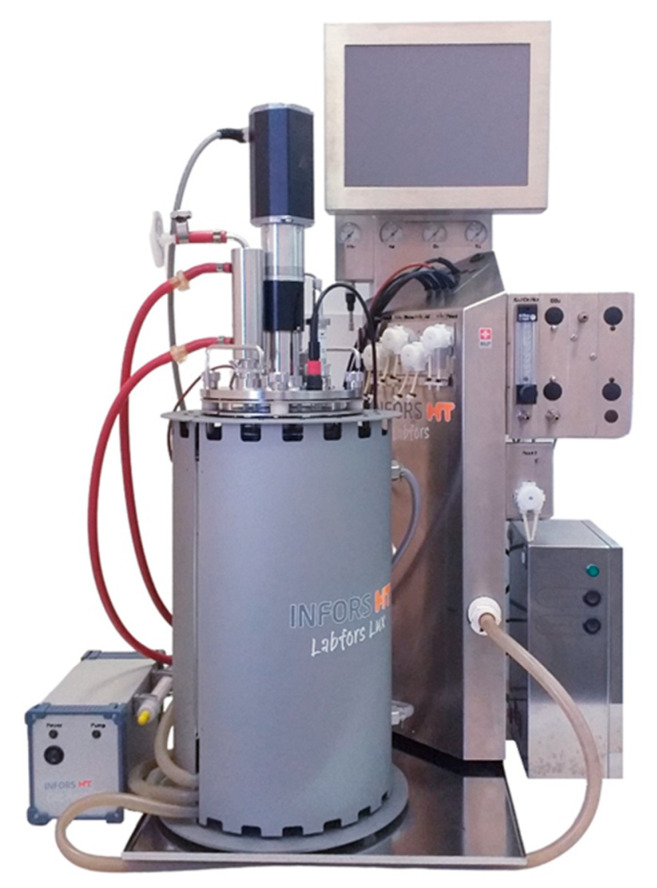
The Labfors 4 Lux photobioreactor (Infors HT, Switzerland) used in this study.

Before starting experiments in a photobioreactor, the alga was grown in 250 mL glass Erlenmeyer flasks containing 30 mL of autoclaved standard BBM. The cells were cultivated for five days on the shaker at 120 rpm at 28 °C and under continuous illumination of 200 μmol·m^−2^·s^−1^. The collected inoculum was then transferred to a sterilized 3.6 L Labfors 4 Lux photobioreactor (Infors HT, Bottmingen, Switzerland) with a working volume of 2.4 L with controlled luminous flux levels (Figure 6). An initial OD_750_ (optical density at 750 nm) of 0.01 was achieved.

In these experiments, to select the optimal concentration of digestate, we cultured algal cells at 28 ± 0.5 °C, under illumination at 1200 μmol photons m^−2^·s^−1^, and with sparging of atmospheric air containing 2.0% carbon dioxide. A modified BBM with an ammonium nitrogen source (NH_4_Cl) was additionally used to compare growth efficiency (final concentrations of ammonium, phosphate, and sulfate ions were ~250, ~160, and ~40 mg·L^−1^, respectively). An amount of 1.3 L·min^−1^ aeration was provided by a compressor. The addition of carbon dioxide was provided by a thermal mass flow controller (Vögtlin Instruments, Aesch, Switzerland). Air and carbon dioxide were mixed and then added to the photobioreactor through a 0.45 μm filter. The photobioreactor was continuously stirred at 120 rpm. The light was maintained on a 16:8 light/dark cycle. pH was measured with an EasyFerm Plus PHI K8 200 electrode (Hamilton, USA) throughout the whole experimental period. In experiments with controlled pH, sterilized 8% NaOH or 8% HCl were used. When observing the foam, a sterile 2% solution of antifoam (Antifoam B, Sigma-Aldrich, St. Louis, MO, USA) was added to the reactor. Specific growth rate (day^−1^) and biomass productivity (g·L^−1^·day^−1^) were calculated as described previously [57].

Two independent experiments were performed to test reproducibility, and the results are presented as mean values.

### 3.3. Analytical Methods

During the growth of microalga, samples were taken from the photobioreactor every 16–24 h to determine the growth, the concentration of pigments in cells, pH changes, and to evaluate the efficiency of nutrient removal by *C. sorokiniana* AM-02 under the tested cultivation conditions.

Optical density (OD) at 750 nm (using cell-free culture medium as reference) and the number of cells were measured every 16–24 h as previously described [27]. After each experimental period, the biomass was collected by centrifugation at 5000× *g* for 10 min.

The final biomass yield (dry weight) and volatile solids were analyzed using a drying oven (at 105 °C for 20 h) and a muffle oven (at 550 °C for 2 h), respectively. Chlorophylls *a* and *b*, carotenoids, and total pigments (mg·L^−1^) were determined using dimethyl sulfoxide extraction and optical absorption correlation, as previously described by Wellburn [58].

The concentration of ammonium ions in the medium was determined by the photometric method. Briefly, samples were centrifuged at 10,000× *g* for 5 min. The supernatant was diluted with distilled water, and 100 μL of Nessler’s reagent (Sigma-Aldrich, St. Louis, MO, USA) was added to each tube and mixed. The tubes were kept for 10 min in the dark, and the optical density was measured at 425 nm using a Lambda 35 spectrophotometer (Perkin Elmer, Singapore).

Ion chromatography was also performed to analyze the bioremediation potential of *C. sorokiniana* AM-02 in terms of utilization of phosphate and sulfate ions. Anion concentration was measured using a Dionex ICS-900 Ion Chromatography System (Thermo Fisher Scientific), as described previously [27]. Nutrient removal efficiency was calculated, as described previously [12].

All measurements were performed in triplicate with two replicates of each experiment. Tukey multiple comparison test was used to compare differences (Minitab software version 20.1.0.0).

### 3.4. Bacterial Community Structure Analysis

At the end of each ADE-containing experiment, samples were taken to analyze bacterial communities’ structure (only one biological replicate was investigated). DNA was extracted and purified from samples after centrifugation of 10 mL at 14,000× *g* for 10 min using a FastDNA spin kit (MP Biomedical, Solon, OH, USA), according to the manufacturer’s protocol. Extracted DNA was then quantified with a Qubit 2.0 Fluorometer (Invitrogen, Carlsbad, CA, USA). Primers Bakt_341F (5′-CCT ACG GGN GGC WGC AG-3′) and Bakt_805R (5′-GAC TAC HVG GGT ATC TAA TCC-3′) were used to amplify the bacterial 16S rRNA gene. Negative extraction control samples did not give visible amplicons, and therefore, they were not analyzed further. Each sample was amplified in triplicate (25 cycles). Sequencing was completed by using an Illumina MiSeq Kit v3 (600 cycles) according to the manufacturer’s instructions. Sequencing of the 16S rRNA gene was conducted in duplicate to ensure reproducibility (two technical replicates were obtained). Additionally, we tried to extract DNA from the pretreated ADE (supernatant), but the DNA concentration was low. This did not allow us to amplify the bacterial 16S rRNA gene in the amounts required for further amplicon sequencing.

The obtained sequence data were then analyzed with the Quantitative Insights into Microbial Ecology (QIIME) software package [59]. High-quality 16S rRNA gene sequences were clustered into OTUs (clustering threshold is 97% identity). OTUs representing less than 0.1% of the total reads were also excluded. Alpha diversity indices were assessed on an OTU level. For the taxonomic assignment of bacterial OTUs, the Silva database was used [60].

## 4. Conclusions

Finally, this work explored whether agricultural wastewater obtained after the anaerobic digestion could replace conventional feedstock in biomass production from *Chlorella sorokiniana* AM-02. The results show that agrarian waste materials are a suitable replacement for traditional media; however, the level of nutrients in these media, such as phosphate and sulfate, must also be controlled to maintain adequate growth of green microalgae. Results showed that *C. sorokiniana* AM-02 is capable of active growth, productivity, and utilization of nutrients in a source of low-quality water. Thus, *C. sorokiniana* AM-02 grew well under 10–40% (*v/v*) anaerobic digestion effluent loading, with the highest growth rate being 1.45 day^−1^ obtained at 10% effluent loading (with the addition of phosphate and sulfate ions). The highest biomass productivity of 0.50 g·L^−1^·day^−1^ was obtained with 20% effluent loading. Besides, microalgae biomass can be considered as animal feed additives or fertilizers.

## Figures and Tables

**Table 1:** *C. sorokiniana* culture characteristics and nutrient removal from Bold’s Basal Medium (BBM) and diluted anaerobic digester effluent (ADE).

Treatment	Dry Weight (g·L^−1^)	Volatile Solids (g·L^−1^)	PO_4_^3−^ Removal (%)	SO_4_^2−^ Removal (%)
modified BBM (pH 7)	2.80 ± 0.11 ^b,c^	2.59 ± 0.07 ^b^	69.1 ± 4.2 ^c,d^	81.2 ± 8.8 ^a,b^
modified BBM (pH 8)	2.83 ± 0.14 ^a,b,c^	2.62 ± 0.11 ^b^	71.0 ± 4.9 ^b,c,d^	82.5 ± 10.6 ^a,b^
10% ADE	0.44 ± 0.04 ^e^	0.41 ± 0.04 ^d^	100 ^a^	100 ^a^
10% ADE + P + S	1.88 ± 0.08 ^d^	1.76 ± 0.08 ^c^	49.7 ± 3.6 ^e^	62.5 ± 9.7 ^b^
15% ADE + P + S	2.73 ± 0.12 ^c^	2.56 ± 0.14 ^b^	66.3 ± 4.0 ^d^	75.1 ± 8.5 ^a,b^
20% ADE + P + S	3.35 ± 0.21 ^a,b^	3.12 ± 0.17 ^a^	75.3 ± 3.8 ^b,c,d^	92.5 ± 7.1 ^a,b^
25% ADE + P + S	3.39 ± 0.23 ^a^	3.20 ± 0.20 ^a^	82.2 ± 2.9 ^b,c^	100 ^a^
40% ADE + P + S	2.51 ± 0.09 ^c^	2.36 ± 0.10 ^b^	85.1 ± 2.7 ^b^	77.5 ± 10.6 ^a,b^

Different superscripts indicate differences between the treatments (ANOVA, Tukey method, α = 0.05). Means that do not share a letter are significantly different.

**Table 2:** *C. sorokiniana* culturing characteristics when grown under different conditions.

Treatment	Specific Growth Rate (Day^−1^)	Biomass Productivity (g·L^−1^·Day^−1^)	Maximum Pigments (mg·L^−1^)	Final Pigments (% Dry Weight)
modified BBM (pH 7)	1.50 ± 0.01 ^a^	0.60 ± 0.02 ^a^	80.5 ± 3.0 ^c^	2.73 ± 0.21 ^c^
modified BBM (pH 8)	1.51 ± 0.01 ^a^	0.60 ± 0.03 ^a^	86.9 ± 4.0 ^c^	2.78 ± 0.28 ^c^
10% ADE	1.15 ± 0.03 ^c^	0.09 ± 0.01 ^d^	4.6 ± 0.7 ^e^	1.02 ± 0.06 ^d^
10% ADE + P + S	1.45 ± 0.01 ^b^	0.40 ± 0.02 ^c^	60.9 ± 5.4 ^d^	2.76 ± 0.11 ^c^
15% ADE + P + S	1.06 ± 0.01 ^d^	0.41 ± 0.02 ^c^	95.8 ± 6.4 ^c^	2.97 ± 0.23 ^c^
20% ADE + P + S	1.08 ± 0.01 ^d^	0.50 ± 0.03 ^b^	124.5 ± 5.6 ^b^	3.58 ± 0.31 ^b,c^
25% ADE + P + S	0.82 ± 0.01 ^e^	0.39 ± 0.03 ^c^	160.4 ± 4.2 ^a^	4.49 ± 0.29 ^a,b^
40% ADE + P + S	0.86 ± 0.02 ^e^	0.33 ± 0.01 ^c^	124.1 ± 6.2 ^b^	4.95 ± 0.10 ^a^

Different superscripts indicate differences between the treatments (ANOVA, Tukey method, α = 0.05). Means that do not share a letter are significantly different.

**Table 3:** Alpha diversity of bacterial communities.

Treatment	Observed OTUs	Chao1	Shannon	Simpson
15% ADE + P + S	60	62	3.99	0.86
20% ADE + P + S	59	59	3.64	0.85
25% ADE_1 + P + S	55	55	3.59	0.86
25% ADE_2 + P + S	61	61	3.74	0.85
40% ADE + P + S	61	62	3.69	0.84
